# Canids as pollinators? Nectar foraging by Ethiopian wolves may contribute to the pollination of *Kniphofia foliosa*

**DOI:** 10.1002/ecy.4470

**Published:** 2024-11-19

**Authors:** Sandra Lai, Don‐Jean Léandri‐Breton, Adrien Lesaffre, Abdi Samune, Jorgelina Marino, Claudio Sillero‐Zubiri

**Affiliations:** ^1^ Wildlife Conservation Research Unit, Department of Biology University of Oxford, The Recanati‐Kaplan Centre Tubney UK; ^2^ Ethiopian Wolf Conservation Programme Dinsho Ethiopia; ^3^ Dipartimento di Scienze e Politiche Ambientali Università degli Studi di Milano Milan Italy; ^4^ AL Wild Expedition Mercus‐Garrabet France

**Keywords:** anthophily, canid diet, *Canis simensis*, inflorescence, mutualism, nectar, pollen vectors, red hot poker, therophily

Up to 87% of flowering plant species depend on a wide range of animal species for their pollination (Ollerton et al., 2011). Among mammals, nectivorous pollinator species are principally represented by flying species such as bats and, to a smaller extent, by some marsupials, rodents, primates, and small carnivores (Carthew & Goldingay, 1997; Regan et al., 2015). It has been pointed out that therophily, pollination by non‐flying mammals, may however be more widespread and hold more significance than currently recognized (Carthew & Goldingay, 1997; Goldingay et al., 1991). For example, in Australia, direct experimentation has shown that the brown antechinus (*Antechinus stuartii*) and the sugar glider (*Petaurus breviceps*) are important pollinators of native Proteaceae (*Banksia* spp.) (Goldingay et al., 1991). The mammals involved in pollination are typically small‐ to medium‐sized and often arboreal species, whereas nectar‐feeding carnivoran mammals are much rarer, with only four species of Carnivora among the 343 mammals identified as potential and known pollinators in a 2015 review (Regan et al., 2015). However, examples of carnivore species foraging for nectar, and putatively involved in pollination, continue to be discovered, such as the masked palm civet (*Paguma larvata*), the Cape genet (*Genetta tigrina*), and the Cape gray mongoose (*Herpestes pulverulenta*) (Kobayashi et al., 2019; Steenhuisen et al., 2015). Here, we report the visitation to inflorescences of the Ethiopian red hot poker (*Kniphofia foliosa*) by a large carnivore, the Ethiopian wolf (*Canis simensis*), in the Bale Mountains of southern Ethiopia. Wolves were observed foraging for nectar on *K. foliosa* flowers, which deposited relatively large amount of pollen on their muzzles, suggesting they could contribute to pollination (Figure 1).

**FIGURE 1:**
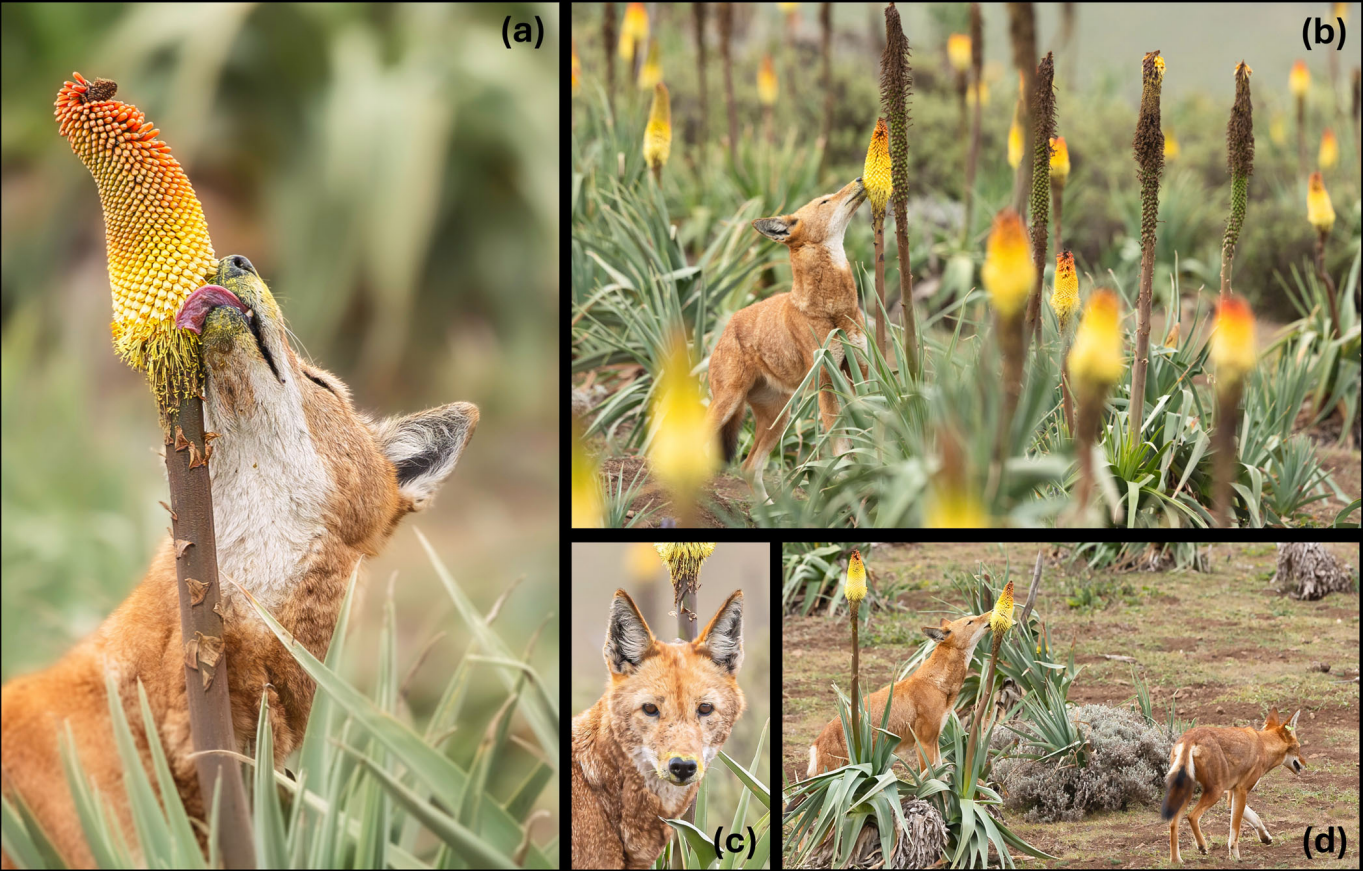
(a) Ethiopian wolf lapping nectar from a *Kniphofia foliosa* inflorescence; (b) nectar foraging in a large *K. foliosa* field of the Web Valley, Bale Mountains National Park, Ethiopia; (c) deposition of a relatively large pollen load on the wolf's muzzle; (d) female adult (left) and female subadult (right) Ethiopian wolves foraging together for *K. foliosa* nectar. See also Video S1. Picture credits: Adrien Lesaffre.

*Kniphofia foliosa* (Asphodelaceae) is a perennial herb endemic to Ethiopia found in the Bale Mountains and other high altitude grasslands (Demissew & Nordal, 2010), which also host the endemic Ethiopian wolf, a top predator restricted to the Afroalpine ecosystem (Marino, 2003). Flowers from the *Kniphofia* genus produce large amounts of nectar, which attracts a variety of bird and insect pollinators (Brown et al., 2009, 2010). The nectar‐feeding behavior of wolves on *K. foliosa* flowers during the main blooming season (June–November; Dagnachew et al., 2022) has been opportunistically but repeatedly observed by the authors over many years. To further detail this behavior, we followed six different wolves foraging on *K. foliosa* inflorescences over four consecutive days in late May‐early June 2023. The observed individuals were one subadult male (<2 years old), four adult females, and one adult of undetermined sex, belonging to three different wolf packs regularly monitored by the Ethiopian Wolf Conservation Programme (Appendix S1: Table S1). Three observations took place in the morning (between 06:22 and 09:29) and three in the afternoon (between 15:27 and 15:59) from an off‐road vehicle at ca. 25 m distance. The total time spent by a wolf moving in a *K. foliosa* flower field varied from 1 min to 1.5 h, during which the time cumulatively spent feeding on inflorescences was between 3 s and 4.5 min. Typically, the wolf approached a stalk and licked the most mature flowers located at the bottom of the inflorescence and containing the most nectar (Figure 1a,b; Video S1). Time spent lapping nectar from an inflorescence ranged between 3 and 15 s. While four wolves visited a few inflorescences (1–5), two visited 20 and 30 inflorescences consecutively during a foray within a flower patch. After feeding on an inflorescence, pollen could clearly be seen deposited on the wolf's muzzle (Figure 1a,c). This behavior highlights the inclusion of nectar in the diet of Ethiopian wolves, but more importantly, it may represent a rare case of potential plant–pollinator interaction involving a large carnivore. Moreover, since these observations covered several individuals from different packs, it indicates that this behavior is not incidental but rather widespread within the population, suggesting its transmission to other individuals potentially through social learning (Figure 1d).

Nectar produced by flowers usually serves as a reward for visitors, drawing in a range of insects, birds, reptiles, or mammals. For the majority of non‐flying mammals, even some small‐bodied ones such as small rodents associated with southern African proteas (Proteaceae), taking nectar from flowers has been regarded as a dietary supplement, insufficient to sustain their energetic needs (the “dessert hypothesis”; Wiens et al., 1983). Some notable exceptions exist among small marsupials, such as the honey possum (*Tarsipes rostratus*), an obligate nectar and pollen feeder (Turner, 1984; Wooller et al., 1999), and the yellow‐bellied glider (*Petaurus australis*), which feeds extensively on the nectar of *Eucalyptus* flowers (Goldingay, 1990). Carnivores feeding on nectar are usually small‐bodied species belonging to the Procyonidae and Viverridae families (all <6 kg in weight; Regan et al., 2015). To the best of our knowledge, the observations we report here highlight the Ethiopian wolf as the only large carnivorous predator documented consuming nectar. Considering Ethiopian wolves' size (12–16 kg; Sillero‐Zubiri & Gottelli, 1994) and specialized rodent diet (Marino et al., 2010), it is unlikely that nectar contributes significantly to their energy budget, tentatively fitting with the dessert hypothesis. Their attraction to the flowers can nevertheless be remarkable, as shown by individuals that sequentially visited 20–30 flowers and dedicated a considerable amount of time to nectar foraging.

This nectar‐feeding behavior raises the question of whether Ethiopian wolves can act as *K. foliosa* pollinators. *K. foliosa* displays floral traits that are considered to promote therophily, notably a large and robust structure, flowers with exserted styles and stamens at anthesis (Appendix S1: Figure S1), and a production of large amounts of nectar and pollen (Carthew & Goldingay, 1997). In *Kniphofia* species, flowers are hermaphrodite, but are predominantly self‐incompatible and thus rely on external vectors such as pollinators to achieve cross‐pollination (Brown et al., 2009, 2010; Duffy et al., 2013). Flower visitation does not by itself represent conclusive evidence that an animal is an effective pollinator (Carthew & Goldingay, 1997). Determining the role of a species as a pollen vector requires a detailed assessment of the frequency of flower visitation, the magnitude of pollen load on the animal, and examining if visits to inflorescences actually result in fruiting (Carthew & Goldingay, 1997; Cunningham, 1991; Goldingay et al., 1991). While our observations indicate clearly that wolves are feeding on nectar and picking up pollen on their fur, it is more difficult to determine and quantify their value as pollinators considering that this mainly depends on their efficiency at transferring pollen to the active stigma of another flower. Pollination effectiveness by mammal vectors might also be confounded by the disturbance resulting from the nectar harvest, since the cost of their damage to flowers might offset any potential contribution to pollination (Fleming & Sosa, 1994; Wiens et al., 1983). Although uncommon, wolves were sometimes observed biting off a few flowers from the inflorescences. Assessing both costs (flower damage, pollen consumption or wastage) and benefits (if and how much outcrossing occurs) is necessary to estimate the value of Ethiopian wolves as functional pollinators for *K. foliosa*.

Furthermore, assessing the importance of Ethiopian wolves as pollinators requires the knowledge of other floral visitors in the system. The community of pollen vectors visiting *K. foliosa* has not been systematically studied, but, as for similar *Kniphofia* species (Brown et al., 2009, 2010), it includes several bird species and insects. Among the avian visitors, we have observed sunbirds (*Nectarinia famosa*, *N. tacazze*), finches (*Serinus negriceps, Crithagra striolata, C. tristriata*), and other passerines (*Pinarochroa sordida*, *Onychognathus morio, Euplectes capensis*, *Ploceus baglafecht*, *Cisticola lugubris*, *Estrilda astrild*). Interestingly, we have also observed other mammals consuming nectar from *K. foliosa*, including humans (mainly children), domestic dogs, mountain nyala (*Tragelaphus buxtoni*), and olive baboons (*Papio anubis*). Since a given flower has limited amount of pollen and nectar but can attract a variety of visitors, the value of a given pollen vector is relative to which other pollinators are available and how they operate (Mitchell et al., 2009; Thomson, 2003). Flying vectors might present higher rates of flower visitation or be more efficient at transferring pollen than terrestrial vectors. In addition, mammals that are only supplementing their diet with nectar may demonstrate a more localized foraging pattern, resulting in a limited range of pollen transfer compared to other vectors (Carthew & Goldingay, 1997). However, there might be a benefit of having both flying and terrestrial vectors as pollinators, since they contribute differentially to plant population structure patterns across the landscape (Wessinger, 2021). The local dispersal of pollen by wolves among plants of a local population may help conserve the genes within that population, whereas more widespread dispersal by flying insects and birds may more effectively disperse pollen among different populations, thus serving as a mechanism for gene flow. The balance between these two vectors may partly determine changes in gene frequency within a local population. Further research will be needed to establish the net benefit to the plant of having wolves as potential pollinators relative to other visitors present in the Afroalpine.

Despite therophily having been reported since the 1930s, there remains a relative lack of studies regarding the reliance of plants on non‐flying mammals for pollination and the selection of specific floral traits facilitating pollination by such mammals (Carthew & Goldingay, 1997; Steenhuisen et al., 2015). With new examples discovered recently, including this present report of a wolf—a large, terrestrial‐bound mammal and otherwise strict carnivore—as a nectar feeder and potential pollinator, an increased awareness of the existence of these lesser‐known pollen vectors could promote research on atypical plant–animal mutualistic interactions. Opportunities for future research in this system include investigating what role *K. foliosa* nectar and pollen play in the wolves' diet, whether wolves are functional pollinators of *K. foliosa* and, if so, their relative importance among the community of floral visitors, and whether there is any evidence of co‐evolution between the wolves and the plants.

## CONFLICT OF INTEREST STATEMENT

The authors declare no conflicts of interest.

## Supporting information


Appendix S1:

Video S1:

Video S1 Metadata:

## Data Availability

Data (Lai, 2024) are available in Figshare at http://doi.org/10.6084/m9.figshare.26125249.
