# Supplementary material for: Canids as pollinators? Nectar foraging by Ethiopian wolves may contribute to the pollination of Kniphofia foliosa
Source: Ecology. 2024 Nov 19;105(12):e4470. doi: 10.1002/ecy.4470 (PMC11610677; doi:10.1002/ecy.4470)
Supplement: Supplementary file 1 — Appendix S1: [file ECY-105-e4470-s001.pdf]

Supporting information. Sandra Lai, Don-Jean Léandri-Breton, Adrien Lesaffre, Abdi Samune, Jorgelina Marino and Claudio Sillero-Zubiri 2024. **Canids as pollinators? Nectar foraging by Ethiopian wolves may contribute to the pollination of *Kniphofia foliosa*.** Ecology.

Appendix S1.

Table S1. Observations of Ethiopian wolves foraging on nectar of *Kniphofia foliosa* in the Web Valley, Bale Mountains, Ethiopia (May-June 2023). Age class consisted of sub-adult (<2 years old; SA) or adult (A); Sex was male (M), female (F) or undetermined (U). Pack membership (3-letter code) was determined from the territory where the focal individual was observed. Wolf ID (3-letter code representing pack membership and unique number) was determined if the animals were ear-tagged using colored and numbered plastic ear tags as part of the long-term monitoring by the Ethiopian Wolf Conservation Programme.

**Date of observation**	**29 May**	**30 May**	**31 May**	**31 May**	**1 June**	**1 June**
Start of observation	07:28	15:27	06:22	15:57	09:29	15:59
End of observation	07:29	16:55	06:22	16:13	09:30	16:23
Pack and wolf ID if tagged	MEG	TAR - TAR22	TAR	TAR	FAT	TAR - TAR02
Age class	SA	A	A	A	A	A
Sex	M	F	U	F	F	F
Number of inflorescences visited	1	30	2	20	2	5
Time spent on an inflorescence	3 sec	3-15 sec	3 sec	5-15 sec	5 sec	4 sec
Total time spent on flowers	3 sec	4.5 min	6 sec	3.3 min	10 sec	20 sec
Behavior before interacting with flowers	Walking	Foraging	Foraging	Foraging	Walking	Foraging

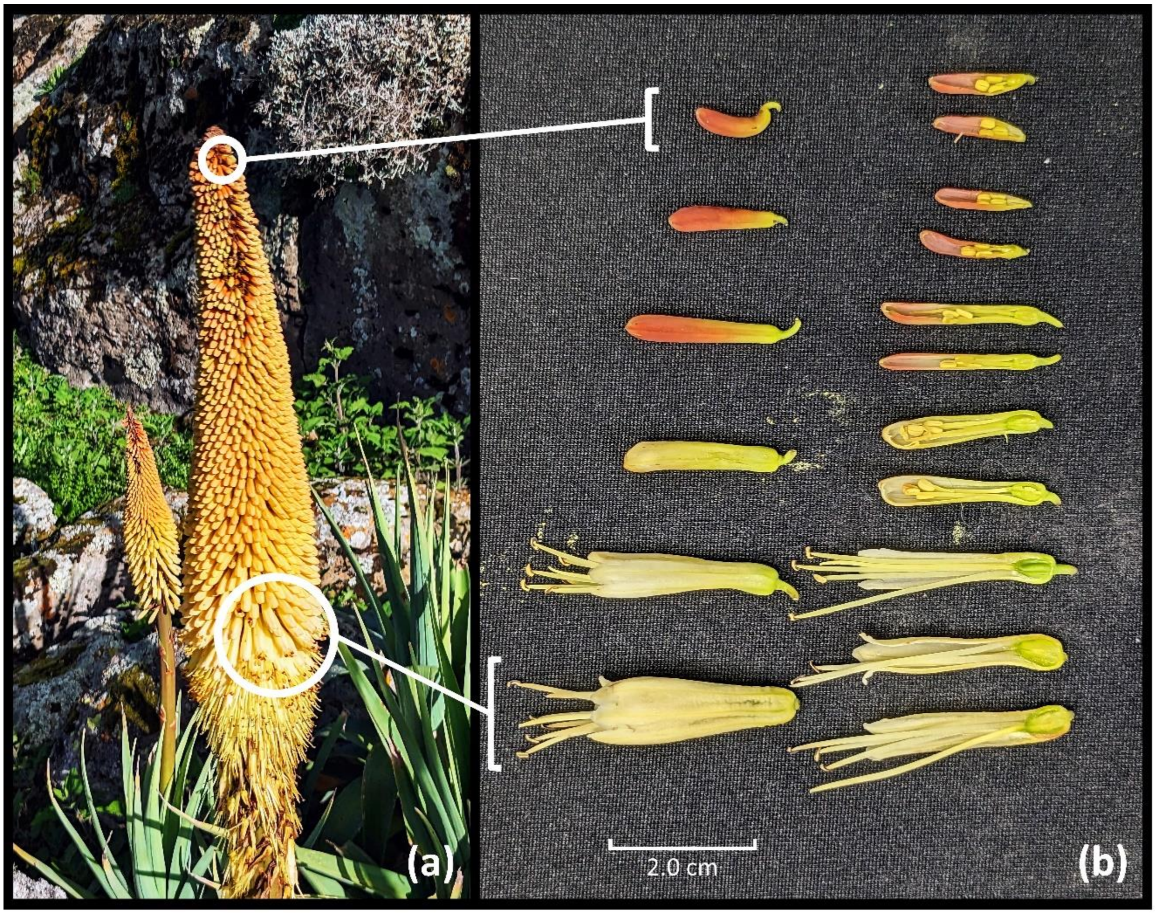

Figure S1. (a) The Ethiopian red hot poker (*Kniphofia foliosa*) features thick erect rhizomes with a basal rosette of sword-shaped leaves and produces elongated inflorescences reaching up to 175 cm in height with dense racemes on a simple erect peduncle. (b) The hermaphrodite flowers of the inflorescence are small and tubular, of shades of vivid red-orange to yellow, with the youngest ones at the apex often more conspicuous, sometimes lending the inflorescence a bicolor appearance. At anthesis, the stamens and style are exserted, with the flower producing copious nectar. Photo credits: Don-Jean Léandri-Breton.
