# Supplementary material for: Canids as pollinators? Nectar foraging by Ethiopian wolves may contribute to the pollination of Kniphofia foliosa
Source: Ecology. 2024 Nov 19;105(12):e4470. doi: 10.1002/ecy.4470 (PMC11610677; doi:10.1002/ecy.4470)
Supplement: Supplementary file 3 — Video S1 Metadata: [file ECY-105-e4470-s002.pdf]

Supporting information. Sandra Lai, Don-Jean Léandri-Breton, Adrien Lesaffre, Abdi Samune, Jorgelina Marino and Claudio Sillero-Zubiri 2024. **Canids as pollinators? Nectar foraging by Ethiopian wolves may contribute to the pollination of *Kniphofia foliosa*.** Ecology.

Video S1. Ethiopian wolf visiting and lapping nectar from three *Kniphofia foliosa* inflorescences, Bale Mountains National Park, Ethiopia, on 30 May 2023. Video credit: Danielle Rubens.
